# Carotid Plaque, Aortic Stiffness, and Left Ventricular Longitudinal Function in Adults with Preserved Ejection Fraction

**DOI:** 10.3390/jcm15218746

**Published:** 2026-11-09

**Authors:** Gheorghe Stoichescu-Hogea, Ana-Maria Pah, Dana Movila, Cristina Savencu, Cristina Ples, Daniel Duda, Simona Ruxanda Dragan

**Affiliations:** 1 Department of Cardiology, “Victor Babeș” University of Medicine and Pharmacy of Timișoara, Eftimie Murgu Square 2, 300041 Timișoara, Romania; gheorghe.stoichescu-hogea@umft.ro (G.S.-H.); man.dana@umft.ro (D.M.); daniel.duda-seiman@umft.ro (D.D.); simona.dragan@umft.ro (S.R.D.); 2 Department of Dental Prostheses Technology, Faculty of Dentistry, “Victor Babeș” University of Medicine and Pharmacy, 9 Revoluției 1989 Blv., 300041 Timișoara, Romania; cristina.savencu@umft.ro; 3 Doctoral School, “Victor Babeș” University of Medicine and Pharmacy, Eftimie Murgu Square 2, 300041 Timișoara, Romania; cristina.stoichescu-hogea@umft.ro

**Keywords:** carotid plaque, arterial stiffness, pulse wave velocity, global longitudinal strain, preserved ejection fraction, vascular–ventricular coupling

## Abstract

**Background/Objectives**: Carotid plaque and aortic pulse wave velocity (PWV) capture distinct aspects of vascular disease. We examined their associations with left ventricular global longitudinal strain (GLS) in adults with ejection fraction ≥50%. **Methods**: This single-center retrospective cross-sectional secondary analysis used the 2018–2019 institutional cohort, comprising preserved-EF ischemic heart disease, hypertension, and reference control groups. The primary outcome was signed GLS, for which a higher (less negative) value indicates worse shortening. Multivariable linear regression simultaneously included plaque and PWV, adjusting for age, sex, systolic blood pressure, diabetes, and source group; HC3 robust confidence intervals were used. **Results**: Of 128 records, 127 had evaluable PWV and GLS. Mean age was 60.8 years; 51/127 (40.2%) were women, and 37/127 (29.1%) had recorded plaque. PWV correlated with GLS (Pearson *r* = 0.426; *p* < 0.001). Adjusted GLS was 0.618 percentage points less negative per 1 m/s higher PWV (95% CI 0.280–0.956; *p* < 0.001). Plaque presence had no clearly detectable independent association (β = −0.348, 95% CI −1.358 to 0.661; *p* = 0.499). **Conclusions**: Higher aortic PWV was associated with less favorable longitudinal function despite preserved ejection fraction. The plaque estimate was imprecise. Cross-sectional data cannot establish temporal order or causality, and the historical ICpFE label does not independently validate an HFpEF diagnosis.

## 1. Introduction

Preserved left ventricular ejection fraction (LVEF) does not ensure normal myocardial mechanics. Diagnosis of heart failure with preserved ejection fraction (HFpEF) requires clinical context and objective evidence beyond LVEF, while global longitudinal strain (GLS) can reveal abnormalities that are not evident from chamber emptying alone [1,2,3,4]. These concepts should be distinguished from the outset. A less negative GLS is an imaging marker of subclinical left ventricular systolic dysfunction that may be present in asymptomatic individuals with hypertension or coronary artery disease, whereas HFpEF is a clinical syndrome that requires symptoms and signs of heart failure together with objective evidence of cardiac structural or functional abnormalities consistent with raised filling pressures, as operationalized by the HFA-PEFF algorithm and the H2FPEF score [1,2]. Abnormal GLS can support, but cannot by itself establish, a diagnosis of HFpEF [3]. We describe the overall sample as adults with preserved ejection fraction and the historical ICpFE group as the preserved-EF ischemic heart disease group, consistent with the prior report from the same cohort [5]. This source-defined group does not establish an independently adjudicated HFpEF diagnosis.

The ventricular–arterial system transmits forward flow and reflected waves across a heterogeneous vascular tree. Left ventricular afterload comprises a steady component, determined mainly by systemic vascular resistance, and a pulsatile component, determined by proximal aortic characteristic impedance, total arterial compliance, and the magnitude and timing of reflected waves [6,7]. Aortic pulse wave velocity (PWV) is the main determinant of reflection timing: as the aorta stiffens, reflected waves return to the proximal aorta earlier, shifting from diastole into late systole and augmenting late systolic pressure, a pattern that predicts incident heart failure [6,8]. Changes in central stiffness can increase late systolic load and accompany impaired longitudinal shortening, although blood pressure, age, sex, and metabolic disease complicate interpretation [9,10,11,12]. Invasive pressure–volume studies have demonstrated combined ventricular systolic and arterial stiffening in patients with HFpEF, and studies integrating central pressure and flow with myocardial geometry have shown that late systolic loading increases myocardial wall stress and is associated with reduced longitudinal (mitral annular) tissue velocities [13,14,15]. Because longitudinal shortening is generated predominantly by subendocardial fibers, which bear the highest wall stress and are most vulnerable to supply–demand mismatch, longitudinal strain may be particularly sensitive to increased pulsatile load [16]. Ventricular–arterial interaction is further modulated by diastolic properties and left ventricular filling pressures, reflected by the E/e′ ratio and left atrial volume, so that the same degree of arterial stiffening may have different ventricular consequences depending on filling conditions [7,17]. Meta-analyses link aortic PWV to later cardiovascular events, but an association with GLS in a selected cross-sectional clinical sample does not imply equivalent prognostic discrimination [18,19,20].

Carotid plaque represents focal atherosclerotic burden, whereas PWV reflects the dynamic propagation of the arterial pressure wave. Prior studies have connected arterial stiffness, ventricular–arterial coupling, and strain, and have examined carotid structure as a vascular risk marker [21,22,23,24,25,26,27,28,29,30]. The association between arterial stiffness and GLS has already been described in hypertensive and community-based populations, and a longitudinal community study showed that progression of arterial stiffness was associated with worsening GLS [10,24,26,27,31]; on its own, therefore, a PWV–GLS relation would be confirmatory rather than novel. Less is known about whether focal atherosclerosis carries information on longitudinal function that is distinct from arterial stiffness. There is a biological rationale for such a link: carotid plaque is a marker of coronary atherosclerotic burden, and coronary disease with subclinical ischemia preferentially affects the subendocardial fibers responsible for longitudinal shortening; consistent with this, asymptomatic patients with significant carotid stenosis and preserved LVEF have shown lower GLS than matched controls [16,32]. At the same time, carotid plaque is a focal structural lesion in a conduit artery remote from the proximal aorta and does not by itself materially alter central impedance or reflection timing, whereas aortic PWV is a central, functional property that directly shapes the pulsatile load imposed on the left ventricle with every heartbeat [6]. We therefore expected that any plaque–GLS association would be largely indirect and attenuated after adjustment, whereas the PWV–GLS association would persist. Few studies have examined focal carotid atherosclerosis and aortic stiffness simultaneously, in the same model, in adults with preserved LVEF across different clinical phenotypes; this is the main contribution of the present analysis. The present analysis tests whether plaque presence and aortic PWV each contribute to variation in GLS among adults with preserved LVEF, with particular attention to the different clinical groups in the clinical database.

## 2. Materials and Methods

### 2.1. Study Design, Source, and Participants

This single-center retrospective cross-sectional study is a secondary analysis of the anonymized clinical database from the Institute of Cardiovascular Diseases Timișoara, Romania. Participants were hospitalized and evaluated during 2018–2019 within the institutional research program. The same parent cohort has previously been analyzed for body mass index and LDL cholesterol [5]; the present analysis addresses distinct vascular exposures and left ventricular longitudinal function and is not an independent replication cohort. The database comprised 128 adults across three source-defined groups: 62 in the preserved-EF ischemic heart disease group (historical database label “ICpFE”), 46 with hypertension (“HTA”), and 20 reference controls (“CONTROL”). All available records had LVEF ≥50%. The reference controls were database-defined and were not independently verified as healthy through standardized prospective screening. Group membership was inherited from the original database, and contemporary HFpEF criteria could not be independently adjudicated. No a priori sample-size calculation was performed. One control record had PWV stored as a date rather than a numeric measurement and was excluded from analyses requiring PWV, leaving 127 participants. Direct identifiers were omitted from the analytical dataset.

### 2.2. Measurements and Definitions

Carotid plaque presence was extracted from the binary plaque field (present versus absent). Plaque was defined according to the Mannheim consensus as a focal structure encroaching into the arterial lumen by at least 0.5 mm or by 50% of the surrounding intima–media thickness (IMT), or demonstrating a thickness >1.5 mm measured from the media–adventitia interface to the intima–lumen interface [33]. Only plaque presence was recorded in the database; plaque number, thickness, area, composition (echogenicity or calcification), and laterality were not captured. Aortic PWV was measured with an oscillometric arteriograph device (TensioMed, Budapest, Hungary), which derives aortic PWV from the return time between the early systolic wave and the reflected late systolic wave recorded by an upper-arm cuff inflated to suprasystolic pressure, using the jugulum–symphysis distance measured over the body surface as the estimate of aortic path length [34,35]. Measurements followed the manufacturer’s recommendations, and recordings were accepted when the standard deviation of PWV across the analyzed cardiac cycles was below 1.0 m/s. Because arteriograph PWV is derived from a brachial waveform and an estimated path length, it is not interchangeable with tonometric carotid–femoral PWV, the reference standard [36]. Left ventricular GLS was extracted as a signed percentage: a higher, less negative value indicates less longitudinal shortening. GLS was obtained by two-dimensional speckle-tracking echocardiography on a GE Vivid ultrasound system with offline analysis in EchoPAC software (version 204; GE Healthcare, Horten, Norway), using apical four-, two-, and three-chamber views and an 18-segment model, and is reported according to the definitions of the joint European Association of Cardiovascular Imaging/American Society of Echocardiography/Industry Task Force on deformation imaging [37]. Acquisition frame rates and intra- and inter-observer variability were not archived for this cohort. LVEF was taken from the recorded planimetric measurement. Indices related to diastolic function and filling conditions available in the database were mitral inflow E and A velocities (E/A ratio), left atrial (LA) volume, and echocardiographically estimated pulmonary artery systolic pressure (PASP). Tissue Doppler e′ velocity, and therefore the E/e′ ratio, and peak tricuspid regurgitation velocity were not recorded, and LA volume could not be indexed to body surface area because height and weight were missing for most participants; diastolic dysfunction therefore could not be graded according to current recommendations [17]. Age, sex, admission systolic blood pressure, diabetes status, and source-defined group were extracted for adjustment. Admission diastolic blood pressure was used to calculate brachial pulse pressure. Use of renin–angiotensin system inhibitors (angiotensin-converting enzyme inhibitors or angiotensin receptor blockers), beta-blockers, statins, and calcium channel blockers was recorded as a binary variable. Right and left IMT values were converted to numeric values when possible and used descriptively. The archived analytical materials do not contain carotid image adjudication, strain acquisition frame rates, or reproducibility data; these limitations constrain measurement reproducibility and comparison with external studies [38,39,40,41,42,43].

### 2.3. Statistical Analysis

Normality of continuous variables was assessed with the Shapiro–Wilk test and Q–Q plots. Because most continuous variables deviated from normality in at least one group, they were summarized and compared with nonparametric methods; GLS, the primary outcome, was approximately normally distributed in the overall sample (Shapiro–Wilk *p* = 0.60), so mean GLS is retained for descriptive contrasts. We report continuous variables as median (interquartile range) and categorical variables as *n*/*N* (%). Differences across the three source-defined groups were compared with the Kruskal–Wallis test for continuous variables and with the Fisher–Freeman–Halton exact test for categorical variables. Pairwise comparisons used Dunn’s test for continuous variables and Fisher’s exact test for categorical variables, with Holm adjustment for multiple comparisons. These between-group comparisons are unadjusted and were not used to infer independent disease effects. Pearson and Spearman coefficients summarize PWV–GLS association. The primary analysis regresses signed GLS on plaque presence and aortic PWV, first jointly and then with age, female sex, systolic blood pressure, diabetes, and indicator variables for hypertension and preserved-EF ischemic heart disease (reference controls as the reference category). We report OLS coefficients with heteroskedasticity-consistent HC3 standard errors and two-sided 95% Wald intervals; *p* values are descriptive. A sensitivity model additionally adjusts for LVEF. Three further sensitivity models added (i) renin–angiotensin system inhibitors, beta-blockers, statins, and calcium channel blockers; (ii) E/A ratio, LA volume, and PASP as available surrogates of diastolic function and filling conditions; and (iii) brachial pulse pressure in place of systolic blood pressure as a simple marker of pulsatile load. Regression residuals were examined with the Shapiro–Wilk test and Q–Q plots; because they deviated mildly from normality, the robustness of the primary model was assessed with nonparametric bootstrap confidence intervals (5000 resamples, percentile method), median (quantile) regression, and a rank-based model in which GLS and PWV were replaced by their ranks. All covariates in these models were present in the *n* = 127 analytic sample, except the E/A ratio, which was missing for one participant. No imputation, automated variable selection, or causal interpretation was applied. Because no a priori sample-size calculation was performed, we did not rely on observed (post hoc) power, which is a direct function of the observed *p* value [44]; instead, we report the minimum adjusted plaque–GLS difference detectable with 80% and 90% power at a two-sided α of 0.05, calculated as (1.96 + 0.84) × SE and (1.96 + 1.28) × SE, respectively, where SE is the HC3 standard error of the plaque coefficient. Analyses used Python (version 3.11.5) with NumPy (version 1.26.0), pandas (version 2.1.1), SciPy (version 1.11.3), statsmodels (version 0.14.0), and scikit-posthocs (version 0.7.0). Version information for this specific secondary analysis was not retained in the supplied analytical materials. Reporting follows observational-study guidance [45].

### 2.4. Ethics and Consent

The study was conducted in accordance with the Declaration of Helsinki. The parent cohort was approved by the Research-Development Ethics Committee of the Institute of Cardiovascular Diseases Timișoara (approval no. 1432, 20 February 2019). The present work is a secondary analysis of the approved retrospective study dataset. Written informed consent, including consent for the use of anonymized clinical and laboratory data for research purposes, was obtained from all participants at hospital admission in accordance with the institutional ethics approval. Direct identifiers were excluded from the analytical dataset and all displays.

## 3. Results

### 3.1. Sample and Vascular Characteristics

The flow from 128 records to 127 evaluable participants is shown in Table 1. Three comparison records had body mass index stored as dates, and one preserved-EF ischemic IMT value contained a double decimal; these fields were treated as missing when summarized. All participants retained for the primary model had numeric LVEF ≥50%, plaque status, PWV, GLS, age, sex, systolic pressure, and diabetes status. Baseline characteristics by source group appear in Table 2.

The preserved-EF ischemic group was older than the comparison group (median 63.0 [IQR 60.0–69.0] versus 52.0 [42.5–60.5] years; *p* < 0.001) and had a higher admission systolic pressure (median 135.0 [120.0–150.0] versus 130.0 [120.0–131.0] mmHg; *p* = 0.017). Diabetes was recorded in 15/62 (24.2%) preserved-EF ischemic and 16/46 (34.8%) hypertension records, but in no comparison records (overall *p* = 0.006). The comparison group therefore differs substantially in clinical profile; unadjusted between-group differences should not be read as independent disease effects. Aortic PWV, GLS, mean bilateral IMT, and plaque prevalence also differed across the three groups (all overall *p* < 0.001), whereas LVEF and the proportion of women did not (*p* = 0.263 and *p* = 0.212, respectively; Table 2). Use of renin–angiotensin system inhibitors, beta-blockers, and statins, as well as the E/A ratio, PASP, and pulse pressure, also differed across groups, whereas LA volume and calcium channel blocker use did not; no comparison participant was receiving any of these drugs (Table 2). Figure 1 displays the participant-level distributions of PWV and GLS by group, together with overall and pairwise *p* values. Both PWV and GLS differed between the comparison group and each clinical group, but not between the hypertension and preserved-EF ischemic groups (Figure 1).

### 3.2. Plaque, Stiffness, and Longitudinal Function

Carotid plaque was recorded in 27/62 (43.5%) preserved-EF ischemic participants, 10/46 (21.7%) participants with hypertension, and none of the 19 evaluable comparison participants (Table 2). Median PWV was 10.1 (9.2–10.8), 9.7 (9.3–10.5), and 7.9 (7.4–8.2) m/s, respectively, and median GLS was −17.7% (−19.2 to −16.4), −17.7% (−20.6 to −16.0), and −20.2% (−21.2 to −19.6). Across all 127 participants, Pearson r for PWV and signed GLS was 0.426 (*p* = 6.1 × 10^−7^); Spearman ρ was 0.409 (*p* = 1.8 × 10^−6^). Figure 2 shows the observed distribution; the line is descriptive and unadjusted.

Participants with recorded plaque (*n* = 37) were older than those without plaque (median 66.0 [IQR 62.0–69.0] versus 60.0 [51.0–68.0] years; Mann–Whitney *p* = 0.004). Their median PWV was 9.7 (9.4–10.9) versus 9.7 (8.4–10.4) m/s (*p* = 0.067), and median GLS was −17.5% (−19.2 to −16.7) versus −18.4% (−20.3 to −16.5) (*p* = 0.159). The unadjusted plaque–GLS difference was 0.759 percentage points (95% CI −0.139 to 1.656; *p* = 0.098). This comparison mixes source groups and has not been adjusted for their differing characteristics. Within the preserved-EF ischemic group, mean GLS was −17.61% with plaque and −17.57% without plaque; in the hypertension group, the corresponding means were −18.18% and −18.51%. These descriptive differences have small plaque-positive denominators and are not formal interaction tests.

PWV–GLS correlation was *r* = 0.543 in the comparison group (*n* = 19; *p* = 0.016), *r* = 0.222 in the hypertension group (*n* = 46; *p* = 0.138), and *r* = 0.375 in the preserved-EF ischemic group (*n* = 62; *p* = 0.0026). Corresponding Spearman coefficients, which do not assume normality, were *ρ* = 0.487 (*p* = 0.034), 0.304 (*p* = 0.040), and 0.263 (*p* = 0.039), respectively (Table 3). These exploratory within-group results are shown in Table 3 and Figure 3. A difference among the three *r* values cannot be inferred merely from their individual *p* values.

### 3.3. Adjusted Associations

In the joint model, each 1 m/s higher PWV was associated with GLS 0.757 percentage points less negative (95% CI 0.482–1.031), while the plaque coefficient was 0.380 (95% CI −0.520 to 1.279). After adjustment for age, sex, systolic pressure, diabetes, and source group, the PWV association remained detectable (β = 0.618, 95% CI 0.280–0.956; *p* = 0.0003), whereas the plaque estimate crossed zero (β = −0.348, 95% CI −1.358 to 0.661; *p* = 0.499). Model R^2^ was 0.295. These coefficients and the LVEF sensitivity analysis are summarized in Table 4 and Figure 4.

When LVEF was added to the adjustment set, the PWV coefficient decreased to 0.547 percentage points per m/s (95% CI 0.203–0.891; *p* = 0.0018), while the plaque estimate remained imprecise (β = −0.327, 95% CI −1.313 to 0.658; *p* = 0.515). This sensitivity model used the same 127 records and had R^2^ = 0.336. The PWV association was also stable after additional adjustment for cardiovascular medications (β = 0.612, 95% CI 0.272–0.952; *p* = 0.0004), after adjustment for E/A ratio, LA volume, and PASP (β = 0.578, 95% CI 0.212–0.945; *p* = 0.0020; *n* = 126), and when pulse pressure replaced systolic blood pressure (β = 0.611, 95% CI 0.260–0.962; *p* = 0.0006), whereas the plaque coefficient remained close to zero and imprecise in every sensitivity model (Table 4). With an HC3 standard error of 0.515 for the plaque coefficient, the primary model had 80% power to detect an adjusted plaque–GLS difference of 1.44 percentage points and 90% power for 1.67 percentage points, but only about 49% power for a difference of 1.0 percentage point; smaller plaque effects therefore cannot be excluded. The observed (post hoc) power for the estimated plaque coefficient was approximately 10%, which simply mirrors its non-significant *p* value. Residuals of the primary model showed mild left skew (Shapiro–Wilk *p* = 0.023). The PWV association was robust to this departure from normality: the bootstrap 95% CI was 0.256–0.904, median regression gave β = 0.700 (95% CI 0.303–1.098; *p* < 0.001), and the rank-based model yielded *p* = 0.003, whereas the plaque estimate remained null (bootstrap 95% CI −1.321 to 0.630; median regression β = −0.447, 95% CI −1.607 to 0.712). PWV tertiles were used only as an exploratory display: the mean GLS was −18.95 ± 1.83% for PWV ≤9.2 m/s (*n* = 45), −18.90 ± 2.80% for PWV 9.3–10.2 m/s (*n* = 44), and −16.83 ± 2.77% for PWV >10.2 m/s (*n* = 38). These cutoffs arise from this sample, and their descriptive pattern does not define clinical thresholds (Table 5; Figure 5).

## 4. Discussion

### 4.1. Principal Findings

The primary finding is a positive association between aortic PWV and signed GLS among adults whose recorded LVEF was at least 50%. In the adjusted model, 1 m/s greater PWV corresponded to GLS that was 0.618 percentage points less negative (95% CI 0.280–0.956). Adding LVEF attenuated the coefficient to 0.547 but did not eliminate the observed association. These results suggest that a vascular stiffness measure captures variation in longitudinal myocardial shortening that is not represented by the LVEF threshold alone. Because both measurements were recorded in a cross-sectional dataset, this is an association and cannot identify its direction or mechanism.

A second finding concerns plaque. The adjusted coefficient for binary plaque presence was −0.348 percentage points (95% CI −1.358 to 0.661), giving insufficient precision to distinguish a small adverse association from a small favorable one. The plaque-positive group was older, and the controls had no recorded plaque, making an overall unadjusted comparison particularly sensitive to source-group differences. Within the preserved-EF ischemic group, mean GLS differed by only 0.04 percentage points between plaque categories. That descriptive contrast is based on 27 and 35 participants and cannot establish equivalence. Figure 5 further illustrates that the highest sample-defined PWV tertile had the least negative mean GLS, but these exploratory cutoffs have no clinical validation.

### 4.2. Potential Hemodynamic Mechanisms

A plausible hemodynamic pathway may explain why higher aortic PWV was associated with less negative GLS. Increased aortic stiffness raises proximal characteristic impedance and forward-wave amplitude and accelerates the return of reflected waves, so that reflection reaches the ascending aorta in late systole rather than in diastole [6,7]. The resulting late systolic pressure augmentation increases and prolongs myocardial wall stress during ejection; invasive pressure–volume and noninvasive pressure–flow studies have linked this loading pattern with ventricular–arterial stiffening, higher myocardial stress, and reduced longitudinal tissue velocities, and population data relate it to incident heart failure [8,13,14,15]. A stiff aorta also lowers diastolic pressure, and thus coronary perfusion pressure, while increasing systolic demand [6].

Longitudinal shortening is generated predominantly by subendocardial fibers, which experience the highest transmural wall stress and are the first to be affected by supply–demand mismatch [16]. Subendocardial dysfunction can therefore reduce GLS while circumferential and radial mechanics maintain LVEF, which is consistent with the less negative GLS observed at higher PWV despite LVEF ≥50%. This pathway could not be tested directly in our dataset, which contained no central pressure waveforms, wave separation analysis, characteristic impedance, total arterial compliance, or myocardial wall stress estimates. Brachial pulse pressure, a crude surrogate of pulsatile load, did not account for the PWV–GLS association when it replaced systolic blood pressure in the model.

Left ventricular filling pressures are a further modulator of ventricular–arterial interaction. Late systolic loading impairs relaxation and may raise filling pressures, and elevated filling pressures and LA remodeling may in turn accompany reduced longitudinal function [7,15,17]. In our cohort, the PWV coefficient was only modestly attenuated after adjustment for E/A ratio, LA volume, and PASP. These indices are, however, incomplete surrogates: without the E/e′ ratio, indexed LA volume, or peak tricuspid regurgitation velocity, diastolic dysfunction could not be graded, and filling pressure may still confound or mediate part of the association. Similarly, adjustment for renin–angiotensin system inhibitors, beta-blockers, statins, and calcium channel blockers, which can modify both arterial stiffness and myocardial deformation, changed the PWV coefficient only minimally.

### 4.3. Comparison with Previous Studies

Studies of arterial load and deformation provide a biologically coherent context for our result. Published investigations have described associations between arterial stiffness, myocardial work or longitudinal strain, and ventricular–arterial coupling in hypertensive and mixed cardiovascular populations [10,11,12,24,25,26,27,29,46,47,48,49,50]. Longitudinal community data have further shown that progression of arterial stiffness, assessed with the cardio-ankle vascular index (CAVI), was associated with worsening GLS [31]. Our PWV–GLS finding is therefore confirmatory; its added value lies in estimating this association simultaneously with carotid plaque in the same individuals with preserved LVEF. Ikonomidis and colleagues specifically examined a PWV-to-GLS ratio in hypertension and related it to vascular and functional markers [12]. Our analysis addresses the two measured components differently: it models PWV as a continuous exposure and signed GLS as an outcome, while adjusting for plaque and recorded covariates. A ratio cannot be substituted for this regression coefficient, and our data do not validate a proposed PWV/GLS clinical score.

The source-group plots show that the PWV–GLS relation is not equally evident in every group. The correlation was strongest among 19 comparison participants and weaker among 46 hypertension participants; interpretation is limited by their narrow PWV range, different baseline risks, and sample sizes. Yoshida and colleagues reported sex differences in associations between stiffness and subclinical left ventricular dysfunction [27]. In our adjusted model, female sex was associated with more negative GLS, but we did not prespecify a sex-by-PWV interaction; the observed sex coefficient should therefore not be presented as confirmation of that effect. Larger studies with calibrated PWV and standardized strain acquisition are needed for transportable estimates.

Evidence on carotid plaque should be interpreted in light of the outcome studied. Studies and meta-analyses have linked carotid plaque or IMT to future atherosclerotic events and shown that plaque can carry risk information beyond IMT [38,39,40,41,42,43,51,52]. Those prospective event outcomes differ from our simultaneous imaging outcome, GLS. The lack of a clearly separable plaque–GLS coefficient here does not challenge plaque’s established role as an atherosclerotic risk marker. Nor can this binary field represent plaque area, composition, echolucency, or total burden. Hong and colleagues found lower GLS in asymptomatic patients with ≥50% carotid stenosis and preserved LVEF than in matched controls [32]; our binary field did not grade stenosis severity, and a relation between carotid atherosclerosis and GLS may become apparent only with more advanced, hemodynamically significant disease or with greater coronary involvement. Aortic PWV and plaque characterize different aspects of vascular disease, which may help explain their differing associations with GLS in this dataset.

The preserved-EF ischemic heart disease group must be distinguished from an adjudicated HFpEF population. Contemporary diagnostic approaches incorporate symptoms, cardiac structure, filling-pressure evidence, and often natriuretic peptides or exercise assessment [1,2,3,53,54,55]. The historical database label ICpFE cannot substitute for these criteria. The results apply to this selected population with preserved LVEF; whether the pattern holds in rigorously phenotyped HFpEF remains uncertain.

### 4.4. Strengths

Several features make the analysis informative within its stated scope. Carotid plaque, aortic PWV, and GLS were present in the same participant-level record, allowing their simultaneous assessment. The signed GLS convention is explicit, so a positive coefficient is interpreted correctly as less longitudinal shortening. We retained all evaluable records, documented the single invalid PWV entry and the additional malformed BMI/IMT fields, used the same 127-person sample for primary and LVEF sensitivity models, and reported confidence intervals with heteroskedasticity-consistent standard errors. Participant-level distributions and group-specific displays make the observed overlap and heterogeneity visible instead of relying on group means alone.

### 4.5. Limitations

The study has a retrospective cross-sectional design and no outcome follow-up. Neither temporal ordering nor a causal pathway between stiffness and GLS can be established. Source-group allocation was not randomized or described as consecutive recruitment; the smaller comparison group differs in age, risk factors, and plaque prevalence, leaving scope for selection bias and residual confounding. Coronary artery disease severity, body mass index in three records, renal function, and contemporaneous hemodynamic status could not all be incorporated reliably into a parsimonious model. Medication use was recorded only as a binary variable, without dose, duration, or adherence; although adjustment for the main cardiovascular drug classes did not materially change the PWV estimate, residual confounding by indication cannot be excluded, particularly because no comparison participant was treated. Admission systolic pressure may not reflect usual pressure, and a single PWV measurement may vary with blood pressure at the time of acquisition. A blood-pressure-independent stiffness index such as CAVI would have been informative in this respect; CAVI has been related to long-term prognosis and to subclinical left ventricular dysfunction, including GLS [31,56,57,58], but it was not available in this database. Adjustment for systolic blood pressure, and replacement of systolic blood pressure by pulse pressure, did not materially change the PWV–GLS association.

Aortic PWV was measured with an oscillometric brachial-cuff device that estimates aortic path length from the jugulum–symphysis distance; although this method has been validated against invasive measurements, its values are not interchangeable with carotid–femoral PWV, which limits cross-study comparability [35,36]. Carotid plaque, although defined according to the Mannheim consensus, was recorded only as a binary entry without archived image adjudication, laterality, quantified burden, or composition. GLS was measured on a single vendor platform, but acquisition frame rates and intra- and inter-observer reproducibility were not archived, and inter-vendor differences in GLS limit comparison with studies using other systems [59]. The E/e′ ratio, indexed LA volume, and tricuspid regurgitation velocity were not available, so diastolic dysfunction and filling pressures could not be formally characterized. The analysis contains 37 plaque-positive participants and no plaque-positive controls, so the plaque estimate has limited precision (80% power only for adjusted differences of approximately 1.4 percentage points or larger) and may depend on modeling assumptions. The secondary group correlations and tertile summaries are exploratory and were not multiplicity-adjusted. Finally, LVEF ≥50% alone does not establish HFpEF, and the file does not identify the full source population or external validation cohort. Differences in vascular acquisition and cardiac functional assessment may affect cross-study comparisons [49,50,60].

A prospective study should enroll a clearly defined clinical population, standardize carotid plaque and aortic PWV acquisition, add a blood-pressure-independent stiffness index such as CAVI, blind strain interpretation, include comprehensive diastolic assessment and central pulse wave analysis, and adjudicate HFpEF independently of LVEF. Repeated measurements and clinical follow-up would help address temporality and prognostic value, which cannot be estimated from this dataset.

### 4.6. Clinical Implications

For patients, the main practical message is that a normal LVEF does not exclude subclinical impairment of longitudinal myocardial function, and that this impairment varies with aortic stiffness. In the primary adjusted model, each 1 m/s increment in PWV corresponded to a GLS value that was approximately 0.6 percentage points less negative; a difference of roughly 2 m/s, similar to the gap between the comparison group and the two clinical groups in this cohort, would therefore correspond to about 1.2 percentage points of GLS. This is relevant because less negative GLS has been associated with adverse outcomes independently of ejection fraction, including in patients with heart failure with preserved ejection fraction [61,62].

Aortic PWV is non-invasive, rapid, and relatively inexpensive, and increased arterial stiffness is recognized in current European hypertension guidelines as a marker of hypertension-mediated organ damage [63]. In patients with preserved LVEF, particularly those with hypertension or ischemic heart disease, an elevated PWV could therefore prompt speckle-tracking assessment of GLS, whereas a less negative GLS could encourage attention to arterial stiffness and its modifiable determinants, such as blood pressure and metabolic risk factors. Such combined vascular–ventricular assessment may help refine risk stratification and the intensity of follow-up beyond LVEF alone. These suggestions remain hypothesis-generating: the present cross-sectional data do not show that PWV-guided strain assessment improves diagnosis or outcomes, do not define a PWV threshold for referral, and do not demonstrate that reducing PWV improves GLS. Prospective studies with standardized measurements and clinical endpoints are needed before such an approach can be recommended in routine practice.

## 5. Conclusions

Among 127 adults with LVEF ≥50% in a single-center retrospective cross-sectional cohort, higher recorded aortic PWV was associated with less negative GLS after adjustment for age, sex, systolic blood pressure, diabetes, plaque presence, and source-defined group. The independent association between binary plaque presence and GLS was imprecise. These observations support further standardized investigation of vascular stiffness and longitudinal myocardial function; they do not establish causation, prognosis, or an HFpEF diagnosis. Clinically, PWV and GLS may provide complementary information in patients with preserved LVEF, but whether their combined assessment improves risk stratification requires prospective evaluation.

## Figures and Tables

**Figure 1:**
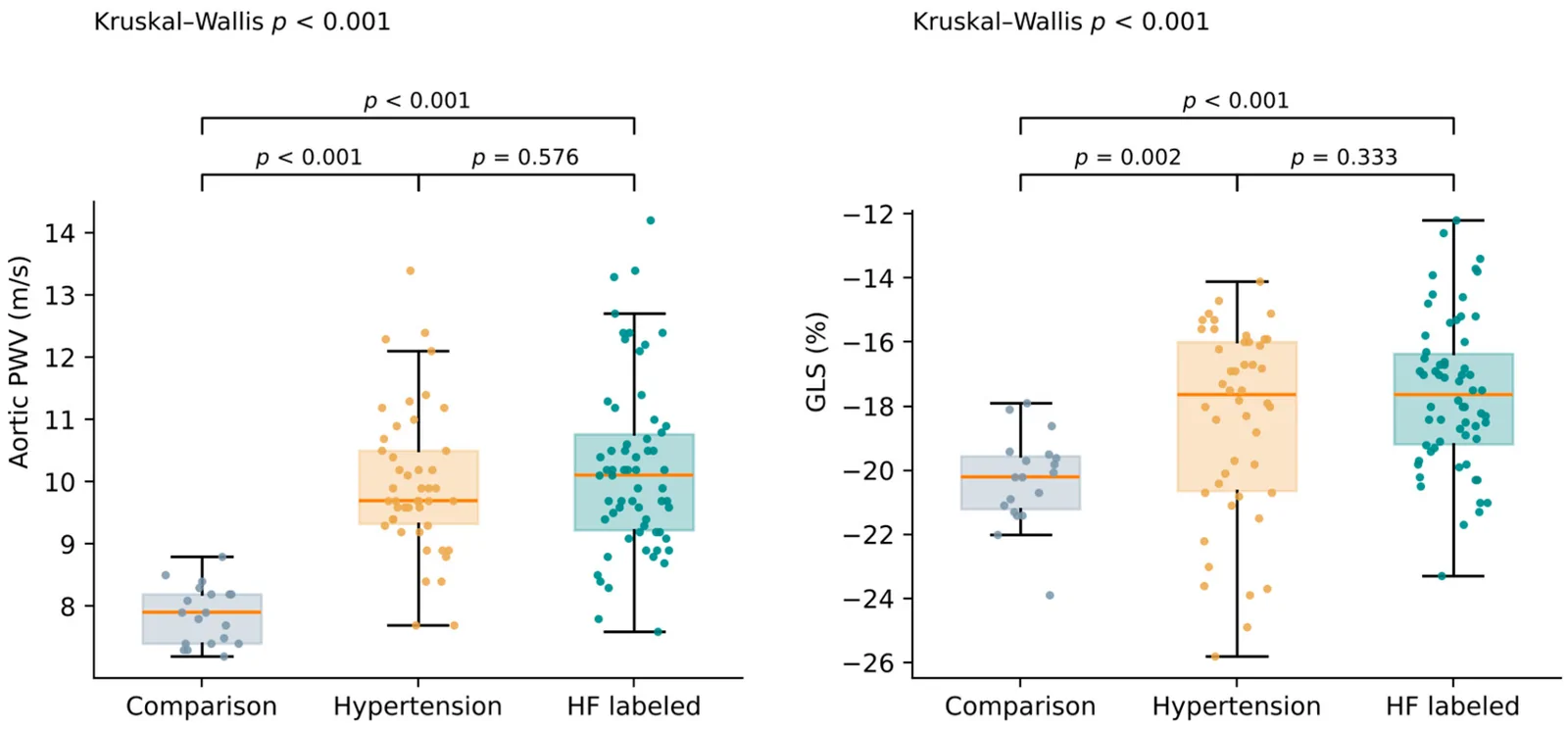
Participant-level aortic PWV and signed GLS by source-defined group. Boxes show median and interquartile range; dots are individual records. Overall *p* values are from the Kruskal–Wallis test; bracketed *p* values are from Dunn’s pairwise comparisons with Holm adjustment. Groups were not matched or randomized, and these comparisons are unadjusted. In the figure, “HF labeled” denotes the historical ICpFE group, described here as preserved-EF ischemic heart disease; “Comparison” denotes reference controls.

**Figure 2:**
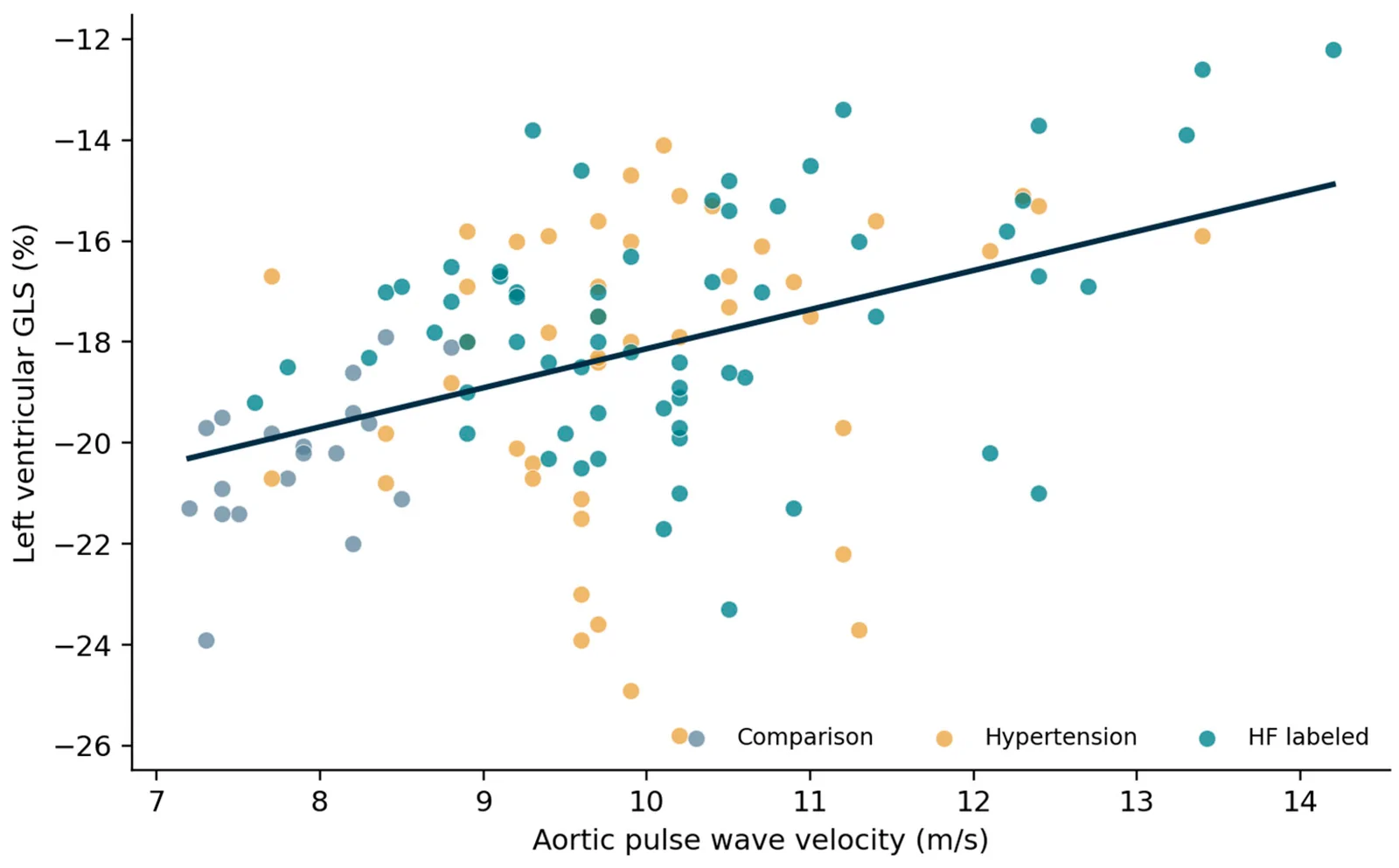
Aortic PWV and signed left ventricular GLS in 127 adults with LVEF ≥50%. Each point is one record; the fitted line is an unadjusted linear trend. More positive GLS values represent less longitudinal shortening. In the figure, “HF labeled” denotes the historical ICpFE group, described here as preserved-EF ischemic heart disease; “Comparison” denotes reference controls.

**Figure 3:**
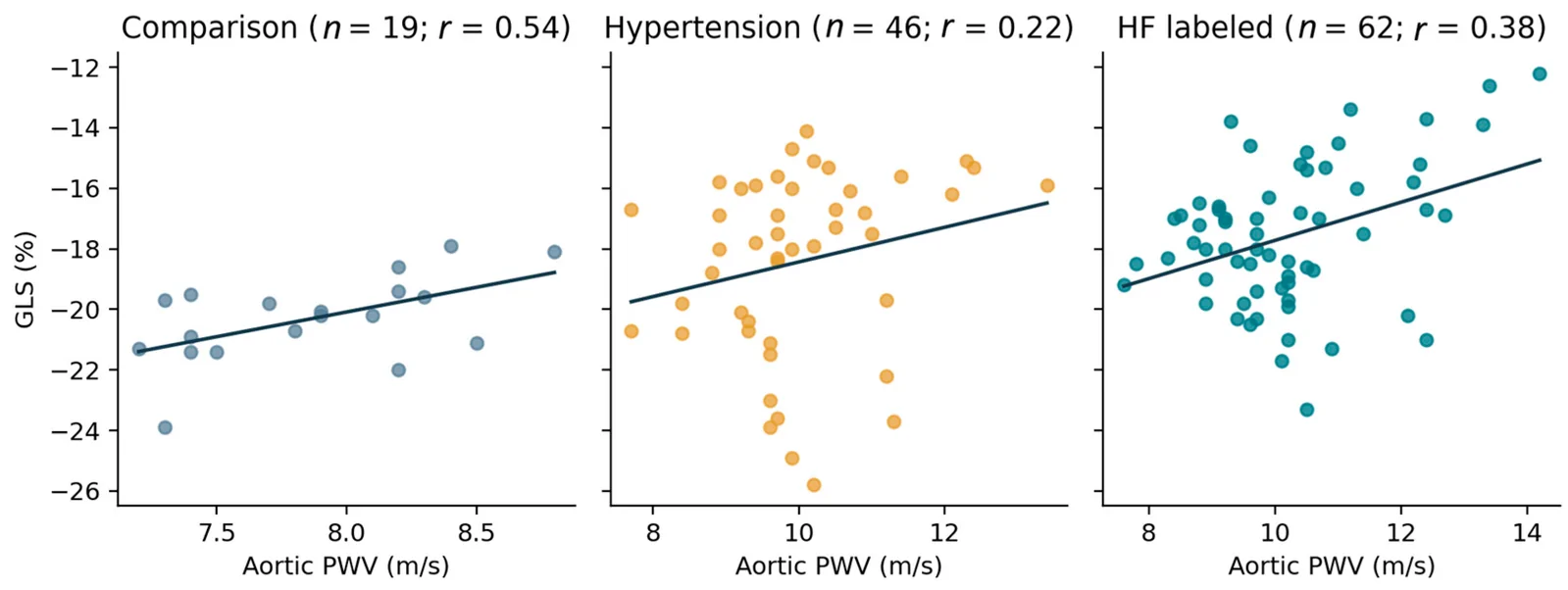
PWV and GLS within each source-defined group. Lines represent separate descriptive least-squares trends. Differences in slopes or correlations were not formally tested. In the figure, “HF labeled” denotes the historical ICpFE group, described here as preserved-EF ischemic heart disease; “Comparison” denotes reference controls.

**Figure 4:**
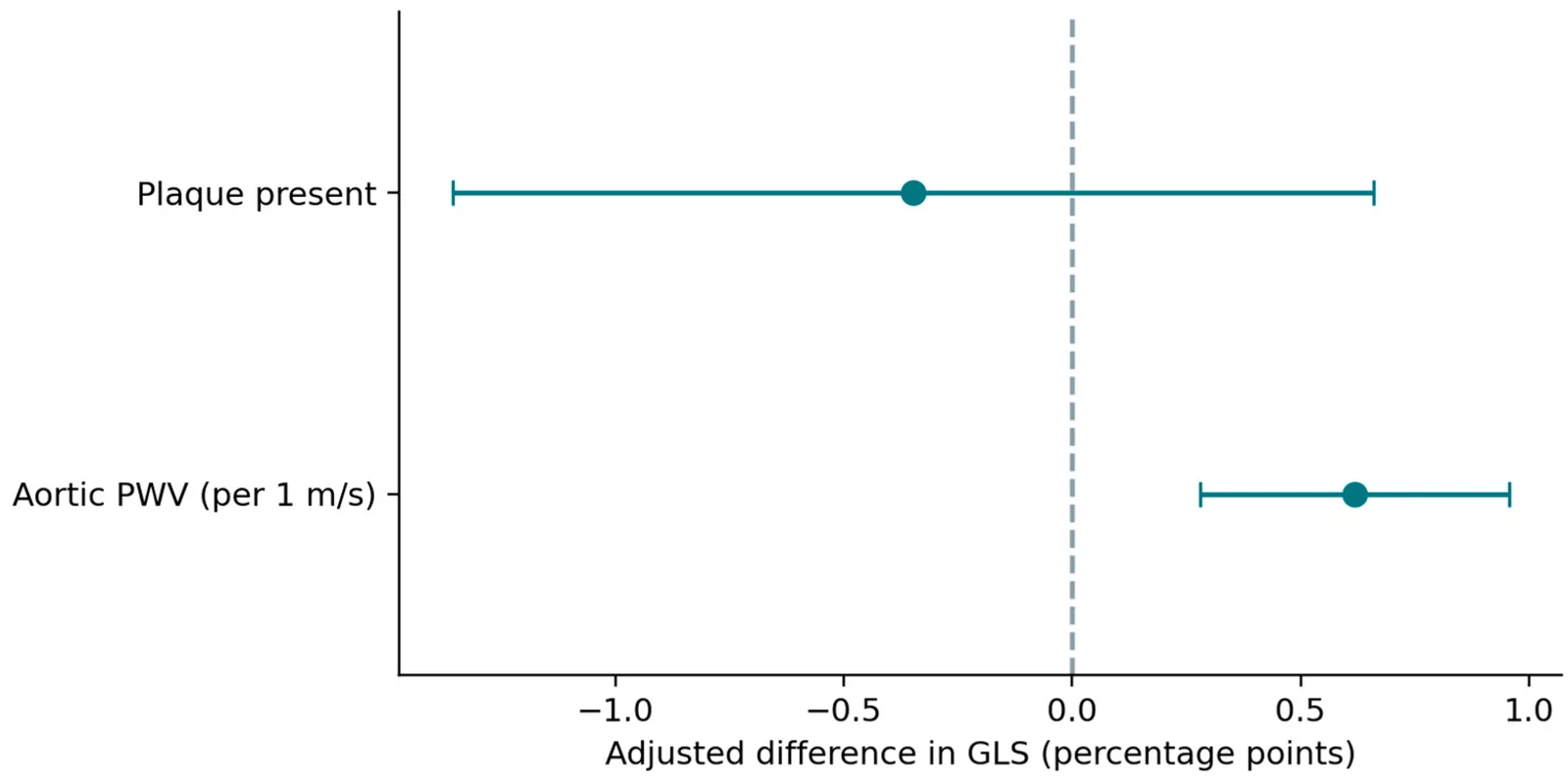
Primary multivariable model (*n* = 127). Points show GLS coefficients; horizontal bars show HC3 95% confidence intervals. A positive PWV coefficient corresponds to less negative GLS.

**Figure 5:**
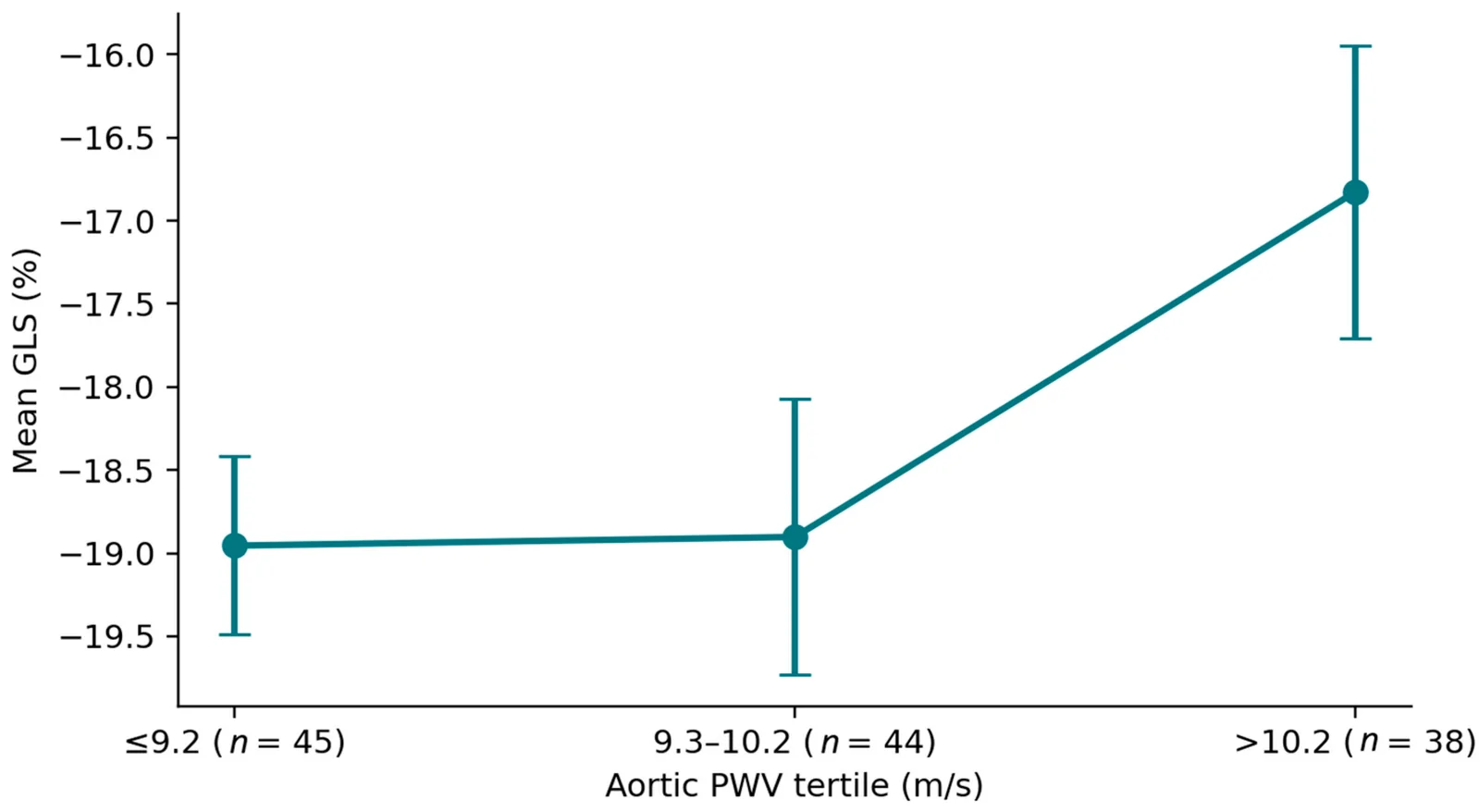
Mean signed GLS by sample-defined PWV tertile, with descriptive 95% intervals calculated as mean ± 1.96 × SD/√*n*. These are unadjusted summaries and do not establish a threshold.

**Table 1:** Participant flow and model-specific sample sizes. Source-defined groups do not independently establish HFpEF.

Step	*n*	Reason/Analytic Implication
Records in the three groups	128	62 Preserved-EF ischemic; 46 hypertension; 20 comparison
Preserved LVEF (≥50%)	128	All records met the LVEF threshold
Primary complete-case sample	127	One comparison PWV stored as date/time; no numeric PWV
Sensitivity model including LVEF	127	Same complete-case sample

EF, ejection fraction; HFpEF, heart failure with preserved ejection fraction; LVEF, left ventricular ejection fraction; PWV, pulse wave velocity.

**Table 2:** Analytic sample by source-defined group (*n* = 127). Values are median (interquartile range) or *n*/*N* (%).

Characteristic	Preserved-EF Ischemic	Hypertension	Comparison	*p* Value
Participants	62	46	19	—
Age (years)	63.0 (60.0–69.0) ^a^	62.5 (53.5–70.8) ^a^	52.0 (42.5–60.5)	0.001
Women	20/62 (32.3%)	22/46 (47.8%)	9/19 (47.4%)	0.212
Diabetes	15/62 (24.2%) ^a^	16/46 (34.8%) ^a^	0/19 (0.0%)	0.006
Renin–angiotensin system inhibitors	29/62 (46.8%) ^a^	15/46 (32.6%) ^a^	0/19 (0.0%)	<0.001
Beta-blockers	30/62 (48.4%) ^a^	17/46 (37.0%) ^a^	0/19 (0.0%)	<0.001
Statins	54/62 (87.1%) ^a,b^	23/46 (50.0%) ^a^	0/19 (0.0%)	<0.001
Calcium channel blockers	11/62 (17.7%)	5/46 (10.9%)	0/19 (0.0%)	0.121
Systolic BP (mmHg)	135.0 (120.0–150.0) ^a^	132.5 (125.0–150.0) ^a^	130.0 (120.0–131.0)	0.018
Pulse pressure (mmHg)	55.0 (50.0–65.0) ^a^	57.5 (50.0–68.8) ^a^	48.0 (40.0–51.0)	0.005
LVEF (%)	55.0 (50.0–55.0)	55.0 (50.0–60.0)	55.0 (55.0–55.0)	0.263
E/A ratio	1.30 (1.14–1.58) ^a,b^	0.83 (0.71–1.23) ^a^	1.50 (1.35–1.59)	<0.001
LA volume (mL)	39.0 (32.3–42.8)	37.0 (34.0–41.8)	36.0 (34.0–38.0)	0.299
PASP (mmHg)	28.0 (25.0–32.0) ^a,b^	31.5 (26.5–35.0) ^a^	24.0 (22.5–26.0)	<0.001
GLS (%)	−17.7 (−19.2 to −16.4) ^a^	−17.7 (−20.6 to −16.0) ^a^	−20.2 (−21.2 to −19.6)	<0.001
Aortic PWV (m/s)	10.1 (9.2–10.8) ^a^	9.7 (9.3–10.5) ^a^	7.9 (7.4–8.2)	<0.001
Carotid plaque	27/62 (43.5%) ^a,b^	10/46 (21.7%) ^a^	0/19 (0.0%)	<0.001
Mean bilateral IMT (mm)	1.05 (0.95–1.25) ^a,b^	0.95 (0.80–1.05) ^a^	0.65 (0.48–0.73)	<0.001

BP, blood pressure; E/A, ratio of early to late diastolic mitral inflow velocity; EF, ejection fraction; GLS, global longitudinal strain; IMT, intima–media thickness; LA, left atrial; LVEF, left ventricular ejection fraction; PASP, pulmonary artery systolic pressure; PWV, pulse wave velocity. Overall *p* values are from the Kruskal–Wallis test (continuous variables) or the Fisher–Freeman–Halton exact test (categorical variables). ^a^ *p* < 0.05 versus comparison; ^b^ *p* < 0.05 versus hypertension (Dunn’s test for continuous variables or Fisher’s exact test for categorical variables, Holm-adjusted). Mean bilateral IMT was available for 61/62 preserved-EF ischemic participants and the E/A ratio for 45/46 participants with hypertension.

**Table 3:** Exploratory within-group correlations. GLS is signed, so positive *r* denotes less negative GLS at greater PWV. No interaction model was prespecified.

Source Group	*n*	Plaque Present	Pearson *r* for PWV–GLS	Descriptive *p* Value	Spearman *ρ* (*p*)
Preserved-EF ischemic	62	27/62 (43.5%)	0.375	0.0026	0.263 (0.039)
Hypertension	46	10/46 (21.7%)	0.222	0.138	0.304 (0.040)
Comparison	19	0/19 (0%)	0.543	0.016	0.487 (0.034)

EF, ejection fraction; GLS, global longitudinal strain; PWV, pulse wave velocity.

**Table 4:** Associations with signed GLS (percentage points), HC3 robust 95% confidence intervals. Primary model adjusts for age, female sex, systolic BP, diabetes, and source group. “Unadjusted separately” contains two independent one-predictor models, with corresponding separate R^2^ values.

Model	Plaque β (95% CI); *p*	PWV β per m/s (95% CI); *p*	*n*; *R*^2^
Unadjusted separately	0.759 (−0.139 to 1.656); 0.098	0.775 (0.517 to 1.034); <0.001	127; 0.017/0.181
Joint plaque + PWV	0.380 (−0.520 to 1.279); 0.408	0.757 (0.482 to 1.031); <0.001	127; 0.185
Primary adjusted	−0.348 (−1.358 to 0.661); 0.499	0.618 (0.280 to 0.956); 0.0003	127; 0.295
Sensitivity + medications	−0.096 (−1.158 to 0.966); 0.859	0.612 (0.272 to 0.952); 0.0004	127; 0.314
Sensitivity + E/A, LA volume, PASP	−0.141 (−1.245 to 0.962); 0.802	0.578 (0.212 to 0.945); 0.0020	126; 0.313
Sensitivity, pulse pressure replacing systolic BP	−0.269 (−1.303 to 0.765); 0.610	0.611 (0.260 to 0.962); 0.0006	127; 0.290
Sensitivity + LVEF	−0.327 (−1.313 to 0.658); 0.515	0.547 (0.203 to 0.891); 0.0018	127; 0.336

BP, blood pressure; CI, confidence interval; E/A, ratio of early to late diastolic mitral inflow velocity; GLS, global longitudinal strain; HC3, heteroskedasticity-consistent covariance estimator type 3; LA, left atrial; LVEF, left ventricular ejection fraction; PASP, pulmonary artery systolic pressure; PWV, pulse wave velocity. Each sensitivity model adds the listed variables to the primary model; medications are renin–angiotensin system inhibitors, beta-blockers, statins, and calcium channel blockers. In the pulse pressure model, brachial pulse pressure replaces systolic BP.

**Table 5:** Exploratory PWV tertiles; observed category sizes differ because tied PWV measurements occur at boundaries. No hypothesis test was prespecified for these categories.

PWV Category	*n*	Mean PWV ± SD (m/s)	Mean GLS ± SD (%)	Plaque Present
≤9.2 m/s	45	8.33 ± 0.63	−18.95 ± 1.83	8/45 (17.8%)
9.3–10.2 m/s	44	9.78 ± 0.29	−18.90 ± 2.80	14/44 (31.8%)
>10.2 m/s	38	11.51 ± 1.02	−16.83 ± 2.77	15/38 (39.5%)

GLS, global longitudinal strain; PWV, pulse wave velocity; SD, standard deviation.

## Data Availability

De-identified clinical and laboratory data supporting the findings of this study are available from the corresponding author upon reasonable request, subject to institutional data-sharing policies.

## Abbreviations

The following abbreviations are used in this manuscript:

BMI	body mass index
BP	blood pressure
CI	confidence interval
E/A	ratio of early to late diastolic mitral inflow velocity
EF	ejection fraction
GLS	global longitudinal strain
HC3	heteroskedasticity-consistent covariance estimator type 3
HFpEF	heart failure with preserved ejection fraction
HTA	historical source label for the hypertension group
ICpFE	historical source label for the preserved-EF ischemic heart disease group
IMT	intima–media thickness
LA	left atrial
LDL	low-density lipoprotein
LVEF	left ventricular ejection fraction
OLS	ordinary least squares
PASP	pulmonary artery systolic pressure
PWV	pulse wave velocity
SD	standard deviation
STROBE	Strengthening the Reporting of Observational Studies in Epidemiology
